# Longitudinal assessment of sweat-based TNF-alpha in inflammatory bowel disease using a wearable device

**DOI:** 10.1038/s41598-024-53522-1

**Published:** 2024-02-03

**Authors:** Robert P. Hirten, Kai-Chun Lin, Jessica Whang, Sarah Shahub, Drew Helmus, Sriram Muthukumar, Bruce E. Sands, Shalini Prasad

**Affiliations:** 1 The Dr. Henry D. Janowitz Division of Gastroenterology, Icahn School of Medicine at Mount Sinai, New York, NY USA; 2 Bioengineering, University of Texas at Dallas, 800 West Campbell Rd., Richardson, TX 75080-3021 USA; 3 EnLiSense LLC, Allen, TX USA

**Keywords:** Predictive markers, Inflammatory bowel disease

## Abstract

Wearable devices can non-invasively monitor patients with chronic diseases. Sweat is an easily accessible biofluid for continuous sampling of analytes, including inflammatory markers and cytokines. We evaluated a sweat sensing wearable device in subjects with and without inflammatory bowel disease (IBD), a chronic inflammatory condition of the gastrointestinal tract. Participants with an IBD related hospital admission and a C-reactive protein level above 5 mg/L wore a sweat sensing wearable device for up to 5 days. Tumor necrosis factor-alpha (TNF-α) levels were continually assessed in the sweat via the sensor, and daily in the blood. A second cohort of healthy subjects without chronic diseases wore the device for up to 48 h. Twenty-eight subjects were enrolled. In the 16 subjects with IBD, a moderate linear relationship between serum and sweat TNF-α levels was observed (R^2^ = 0.72). Subjects with IBD were found to have a mean sweat TNF-α level of 2.11 pg/mL, compared to a mean value of 0.19 pg/mL in 12 healthy controls (*p* < 0.0001). Sweat TNF-α measurements differentiated subjects with active IBD from healthy subjects with an AUC of 0.962 (95% CI 0.894–1.000). A sweat sensing wearable device can longitudinally measure key sweat-based markers of IBD. TNF-α levels in the sweat of subjects with IBD correlate with serum values, suggesting feasibility in non-invasive disease monitoring.

## Introduction

Inflammatory bowel disease (IBD), comprised of ulcerative colitis (UC) and Crohn’s disease (CD), are chronic inflammatory conditions of the gastrointestinal tract^1,2^. Discordant symptoms and inflammation, as well as the unpredictable nature of flares, make IBD challenging to manage^3^. Monitoring modalities include symptom assessment, serum or stool inflammatory marker collection, and imaging or endoscopic evaluations. All modalities are limited to a single point of evaluation, are inconvenient to collect, and can be invasive.

This highlights the need to develop new monitoring modalities that can assess disease markers longitudinally and in real time. Wearable technology is increasingly accepted for the monitoring of chronic disease and health. It enables the passive and continuous measurement of non-invasive physiological metrics^4^. Traditional wearable devices utilize optical and actigraphy sensors enabling assessment of metrics including heart rate, heart rate variability, physical activity and sleep. Metrics collected from optical sensors, such as heart rate variability, have been shown to be associated with symptom and inflammatory markers in individuals with IBD^5^. However, the exploration of new types of wearable devices are needed.

The sweat represents an attractive biofluid to explore for sampling and monitoring using wearable devices, as it can be assessed non-invasively and continually for biomarkers important to health and disease^6–8^. Sweat is a clear fluid that is comprised of approximately 99% water. It is secreted from over 2.5 million sweat glands in the skin which produce approximately 1 quart of sweat per day. However, sampling of sweat poses many challenges which are not present in the collection of other body fluids, such as blood, urine or stool. As an example, sweat concentration varies based on the body location of sampling and can be impacted by variable rates of evaporation. Additionally, the rate of sweating varies over time and between individuals, which may prevent adequate samples from being collected. The skins exposure to the environment increases the risk of sample contamination and can additionally be challenging to sample due to movements and an uneven skin surface^9,10^. Biomarkers from the blood including electrolytes and proteins actively or passively diffuse into sweat in measurable concentrations, with final concentrations impacted further by analyte size, charge, and sweating rate^11,12^. Despite this, the relationship between many sweat analytes with their serum concentrations are not known, with studies limited by a lack of standardized methods for sweat sampling and analysis^13^. Therefore, many sweat based biosensors focus on a limited number of analytes such as electrolytes, glucose and lactate^14,15^. Recent advances have started to explore other analytes in greater detail including essential amino acids and vitamins, as they relate to nutrition assessments^15^. Thus, there is opportunity to assess other analytes such as proteins and other sweat based biomarkers that relate to chronic inflammatory disease activity.

Until recently, the primary means to assess sweat was via an adhesive bandage like patch which has been shown to be effective in assessing markers in small cohorts and in correlating these results with serum measurements^16–18^. However, the primary limitations of using a sweat patch is that it provides data for a limited duration of time such as just a few days, the measurement is not continuous, and only an average value from a relatively long fixed time period is available. Additionally, the analytes collected by the patches need to be extracted in a time-consuming process which doesn’t produce immediately actionable results. Current technological advances focus on real-time monitoring. Most sweat sensors require larger volumes of sweat for analysis requiring stimulation. Stimulation methods range from pressure driven collection, exercise or ionophoresis. However, there have been advancements, including by our group, in sedentary sweat sampling which is able to measure analytes at lower sweat volumes^6,12,19^. Advances in nanotechnology have enabled further advancements in sweat sensing devices utilizing various detection techniques to expand the range of analytes that can be measured. Gao et al. and He et al. developed fully integrated sensors incorporating flexible multiplexed perspiration analyzers and carbon textile hierarchical porous mesh, respectively, for in situ perspiration analysis measuring analytes such as glucose, lactate and electrolytes^20,21^. The same group explored the measurement of heavy metals in the sweat by developing a microsensor array for multiplexed monitoring of metals such as Zinc and Cadmium^22^. Sempionatto et al. explored flexible printable tattoo electrodes immobilized with ascorbate oxidase to monitor changes in vitamin C levels in the sweat^23^. Other advancements include the development of low cost and easy to fabricate devices for the measure of analytes such as a electrolytes^9^.

An area of significant unmet need is the ability to monitor inflammation, and thereby inflammatory based diseases, non-invasively utilizing sweat sensing technologies. Several biosensors have been developed in this space, however most actively stimulate the sweat glands to produce sweat for sensing either by electrical stimulation or exercise, potentially invoking and thereby influencing the levels of inflammatory biomarkers^24,25^. Additionally, most of the biosensors in this area provide only single time point estimates of the analyte^16,17,26,27^. To address many of these limitations yet take advantage of the ability to measure physiologically meaningful analytes in the sweat, our group developed a sweat based wearable device. The IBD AWARE device was the first demonstration of a sweat sensing device to detect inflammation in humans from passively expressed sweat, where it was demonstrated to measure IL-6, IL-8, IL-10 and TNF-alpha in a continuous manner^25^. It enables sedentary sweat sampling over a period of up to 5 days and the real time assessment of analyte concentrations through a blue tooth connection with our custom smartphone app. Furthermore, our sweat sensing platform is fully modifiable, which enables the assessment of up to 4 analytes at one time. This makes it possible to assess various markers or combinations of markers as they relate to IBD or other chronic diseases, in real time, and nearly continuously. This approach creates the potential to develop a novel means to monitor disease activity, or through the assessment of temporal changes of analytes, evaluate novel relationships between disease markers and outcomes. Previously, our group piloted this technology to demonstrate that sweat sensing wearable devices are able to track analytes such as CRP and IL-1β levels in healthy controls for up to 30 h^28^. However, longitudinal assessments of biomarkers are needed to best assess chronic diseases over time. The longitudinal assessment of such markers in patients with IBD is an important next step in the development of such technology for disease monitoring.

**Figure 1:**
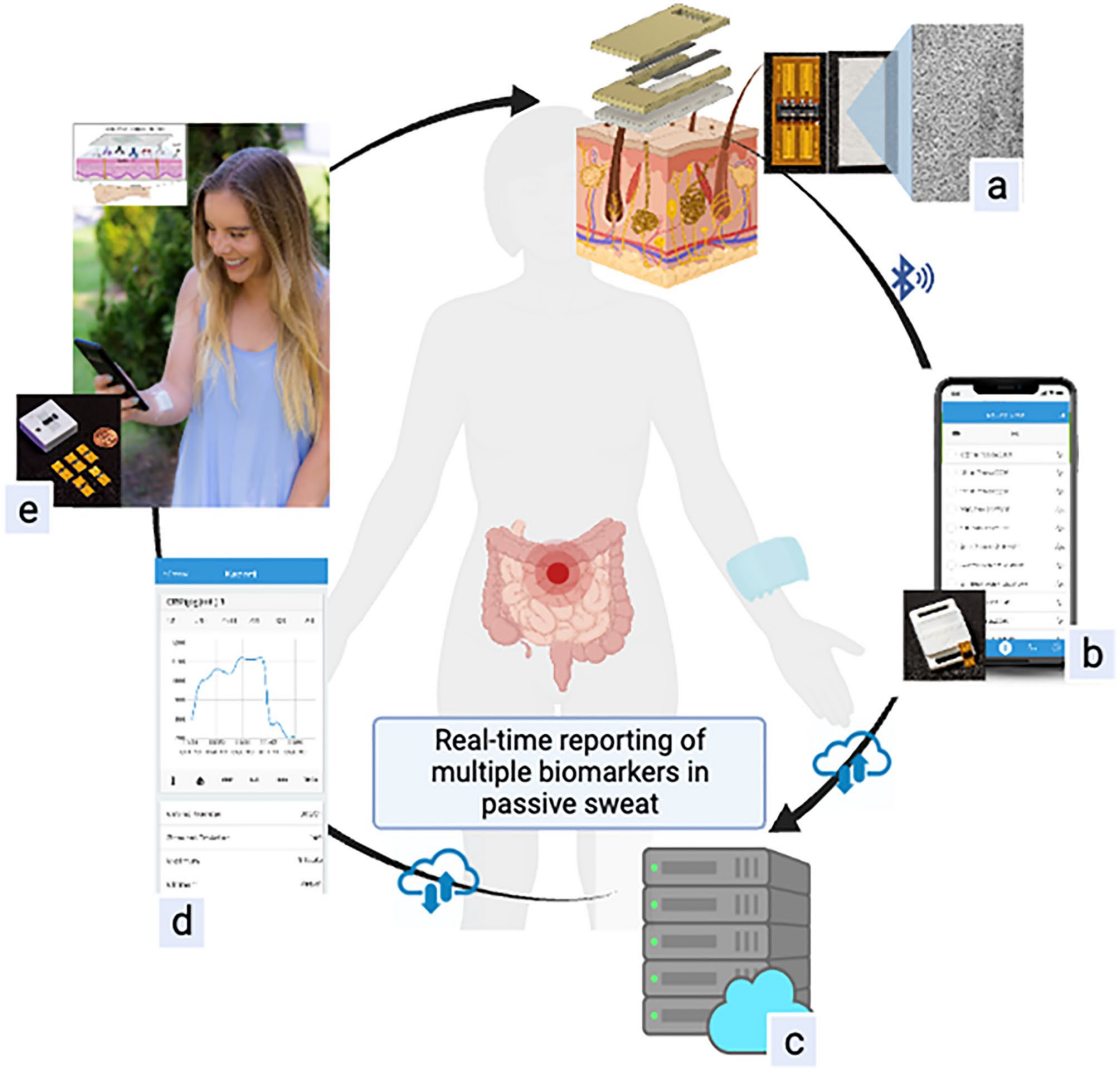
The IBD AWARE device passively collects analytes in the sweat. Data is transmitted in real time via Bluetooth to an individual’s smart phone or tablet and then to the cloud server. This enables the real time reporting of biomarker levels to patients and providers. (**a**) The sensor with pore structure to facilitate sweat absorbance. (**b**) The data is transferred via Bluetooth through a smart phone or tablet. (**c**) The data is uploaded to the cloud and processed on the server. (**d**) Data can be visualized by health care providers or patients via a custom portal. (**e**) Continuous reading of protein analytes in the sweat. The small insert is the illustration of the device and sensor alongside a penny for size comparison. Figure was created with BioRender.com.

Sweat contains many protein biomarkers found in the serum which play an important role in the monitoring and pathophysiology of IBD and other chronic diseases. This includes inflammatory proteins such as C-reactive protein and cytokines such as tumor necrosis factor alpha (TNF-α), interlukin-1, and interlukin-6^25,28–30^. Inflammatory markers and cytokines are released from the intravascular membrane and move into the dermal interstitium subsequently diffusing into the sweat glands before reaching the skin via hydrostatic pressure^27,31^. Several studies have found significant correlations between 24 h measurements of sweat and plasma cytokines, with strong correlations for some markers such as TNF-α (R^2^ = 0.95), and lower correlations for others, such as IL-1 (R^2^ = 0.70)^16,17^. Our group has demonstrated high correlations between sweat and serum cytokines and inflammatory markers for IL-1β (R^2^ = 0.99) and CRP (R^2^ = 0.99)^28^. Proinflammatory cytokines play a key role in IBD, resulting in the activation of the adaptive immune system and in subsequent tissue damage^32^. TNF-α is a pro-inflammatory cytokine that is associated with regulating several inflammatory pathways making it a key mediator in the inflammatory response. Its activation of effector cells may result in apoptosis or aid in the defense against infections or localized tissue injury. Elevated levels of TNF-α in IBD patients results in an irregular proinflammatory response and dysregulated mucosal immune activity^33,34^. While TNF-α is initially a transmembrane protein, it can be cleaved into a soluble form that is measurable in the systemic circulation. Through diffusion it enters the sweat^32,35^. TNF-α levels are elevated in individuals with inflammatory bowel disease compared to healthy controls^36^. Furthermore, it is the primary target of the widely used and effective anti-TNF-α agents, such as infliximab and adalimumab. Thus, given its central role in IBD, it is an attractive marker to evaluate in our pilot assessment of a sweat sensing device in this population. The goal of our current study seeks to build on our prior work evaluating a novel sweat sensing device in healthy individuals (Fig. 1). Here we perform a longitudinal assessment of how key markers of IBD activity, in this case TNF-α, are correlated between the sweat and serum of subjects with IBD. Furthermore, we assess whether expected differences in serum concentrations between subjects with active IBD and healthy controls are reflected in the sweat.

## Results

Sixteen subjects with IBD were enrolled between November 2021 and August 2022 (Table 1). They consisted of 4 subjects that had CD, 10 subjects that had UC, and 1 subject with indeterminate colitis. The median age of subjects was 41.5 years, and 37.5% of subjects (6/16) were men. Participants were followed for a median of 4 days. Clinical disease activity indices were reported each day (Supplemental Table 1). In a separate cohort, 12 healthy subjects were enrolled with a median age of 28.5 years (Supplemental Table 2).

**Table 1:** Characteristics of subjects with inflammatory bowel disease.

	Cohort, n = 16 (%)
Age, years, median	41.5
Male sex	6 (37.5)
Race	
White	7 (43.75)
Black	5 (31.25)
Asian	2 (12.5)
Prefer not to say	2 (12.5)
Ethnicity	
Hispanic	1 (6.25)
Not Hispanic	14 (87.5)
Prefer not to say	1 (6.25)
Smoking History	
Current	3 (18.75)
Former	3 (18.75)
Never	10 (62.5)
Ulcerative colitis disease location	
E1	0 (0)
E2	5 (45.45)
E3	6 (37.5)
Crohn’s disease location	
Ileum only	0 (0)
Colon only	3 (50)
Ileum and colon	2 (33.33)
Upper gastrointestinal tract	1 (16.67)
Previous IBD medications	
Mesalamine	6 (37.5)
Mercaptopurine, azathioprine or methotrexate	3 (18.75)
TNF-α	4 (25)
Vedolizumab	1 (6.25)
Ustekinumab	1 (6.25)
Tofacitinib	1 (6.25)
IBD medications at enrollment	
Mesalamine	1 (6.25)
Mercaptopurine, azathioprine or methotrexate	2 (12.50)
TNF-α	3 (18.75)
Vedolizumab	4 (25)
Ustekinumab	0 (0)
Tofacitinib	0 (0)

E1: proctitis (proximal extent to the sigmoid colon), E2: left-sided disease (to the splenic flexure), E3: extensive disease (beyond the splenic flexure).

### Serum and sweat TNF-α correlations

**Figure 2:**
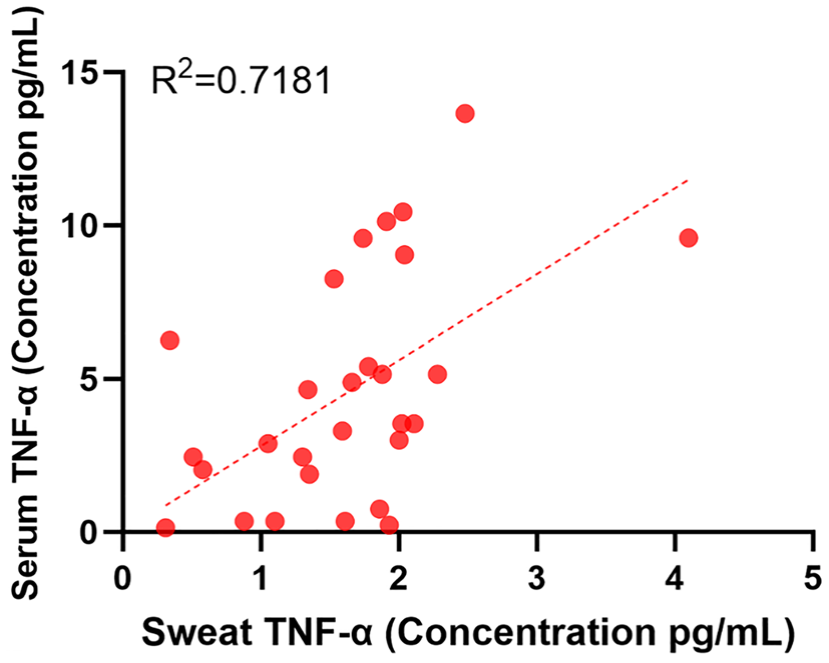
Correlation between serum and sweat TNF-α levels.

In subjects with IBD, the IBD AWARE device measured TNF-α in the range of 0.31–4.1 pg/mL. All serum to sweat ratios were plotted for the 11 subjects with IBD whose serum and sweat measurements for TNF-α overlapped by  ± 30 min (Supplemental Table 2). Twenty-eight days’ worth of data were plotted. The linear correlation coefficient between the two metrics was R^2^ = 0.72 (Fig. 2).

### Clinical classification and temporal relationships

**Figure 3:**
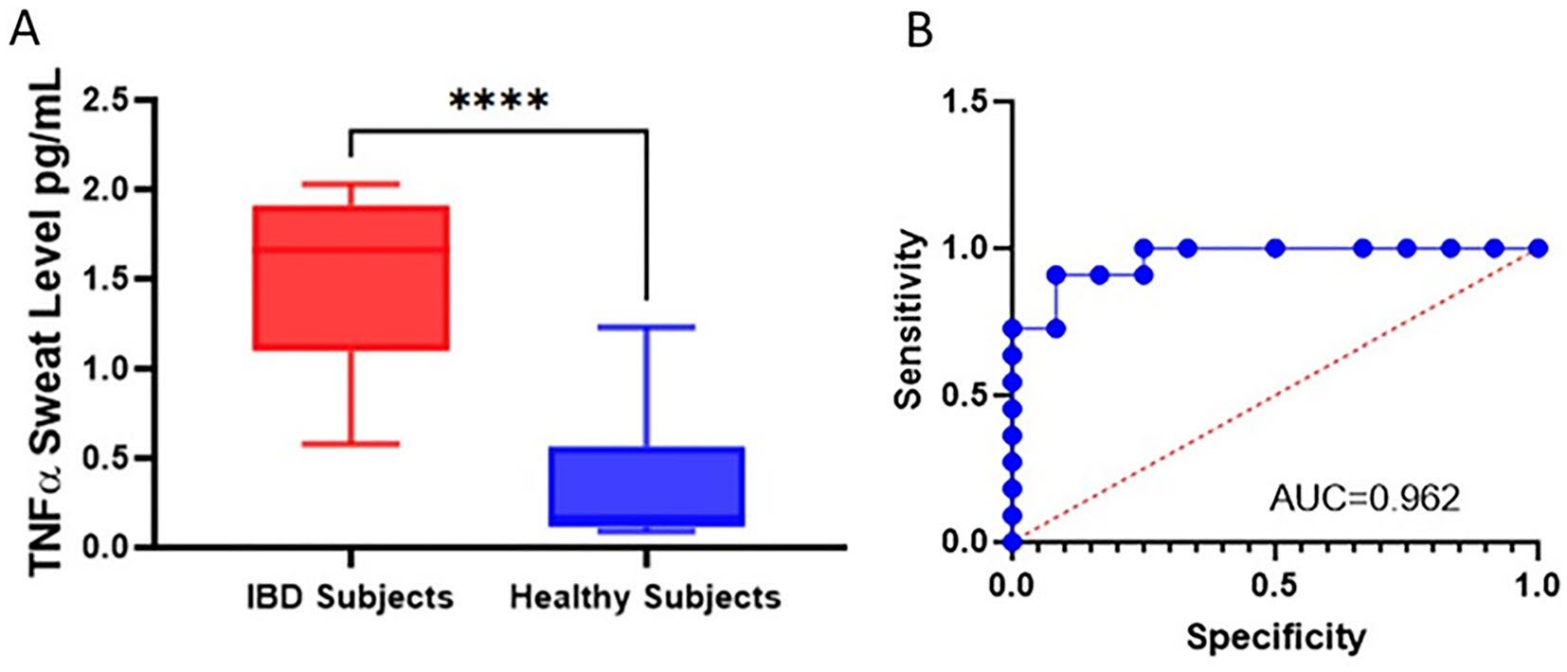
The difference between sweat TNF-α levels in subjects with active IBD and healthy controls. (**A**) The mean TNF-α levels in subjects with IBD and healthy controls are compared using a t-test (*p* < 0.001). (**B**) The Receiver Operative Characteristic (ROC) curve for sweat TNF-α with the computed area under the curve (AUC = 0.962).

**Figure 4:**
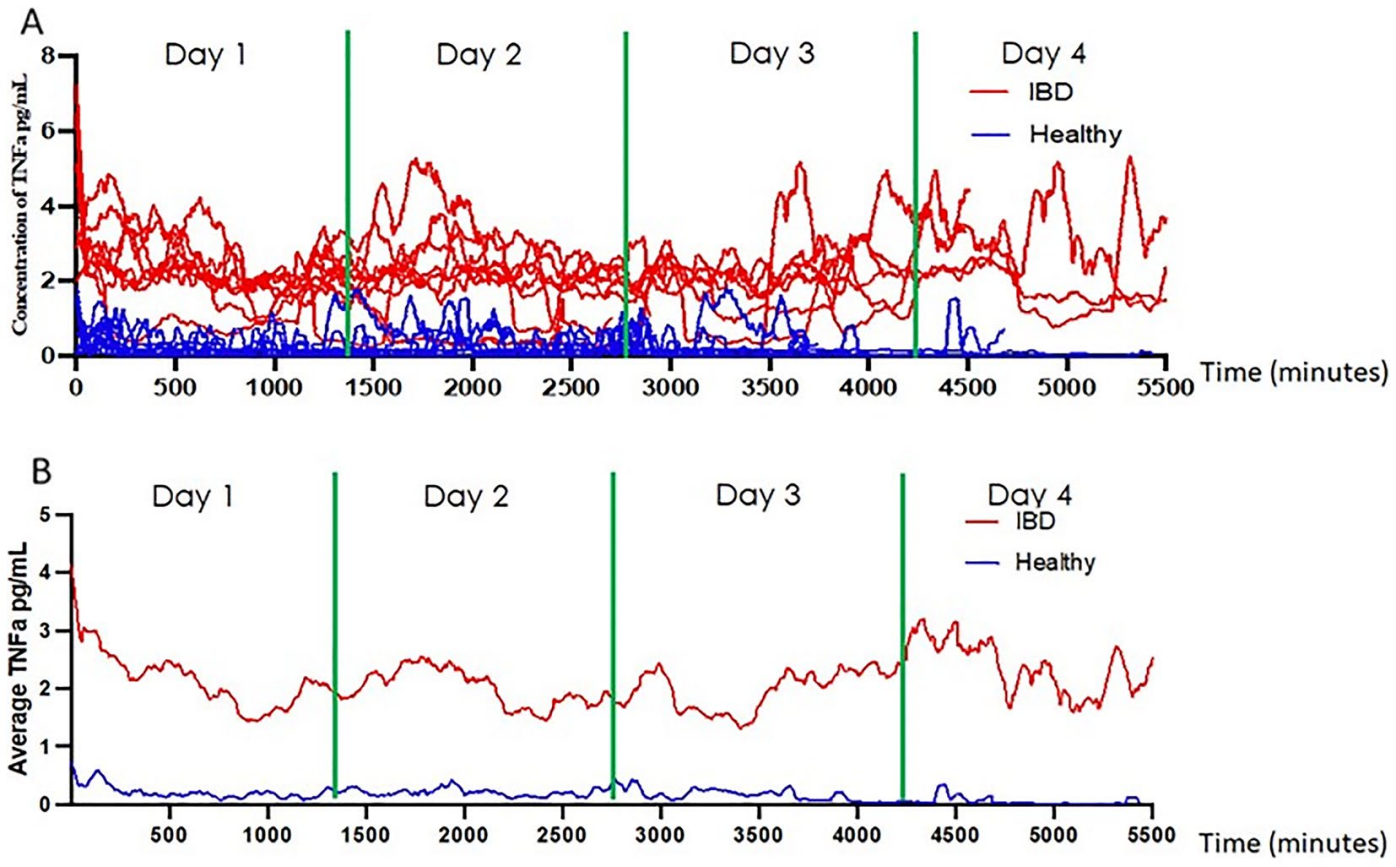
Temporal plots of sweat TNF-α levels. (**A**) Sweat TNF-α levels are plotted at 1 min intervals in subjects with IBD (red) and healthy controls (blue). Each line represents a subject’s sweat values over the follow up period. (**B**) The mean sweat TNF-α level for the cohort of subjects with IBD (red) and healthy controls (blue) over the follow up period.

Sweat TNF-α levels were compared between subjects with active IBD and healthy controls. The IBD AWARE device measured TNF-α in the sweat of healthy controls in the range of 0.09–1.23 pg/mL. The mean sweat TNF-α level for subjects with IBD, in a  ± 30 min window around serum measurements, was 1.541 pg/ml (std = 0.46), while in healthy controls it was 0.3508 pg/ml (std = 0.37), over a 1 h window approximately 30–60 min after applying the device in the morning. As seen in Fig. 3A, there was a significant difference between sweat TNF-α levels in subjects with active IBD compared to healthy controls (*P* < 0.0001). To further support the observation that there is a significant difference between serum and sweat levels of TNF-α in those with and without IBD, the ROC was plotted (Fig. 3B). In agreement with this finding, we observed an AUC of 0.962 (95% CI: 0.894–1.000) and a sensitivity and specificity of 90.9% and 91.7% at a TNF-α cutoff of 0.98 pg/mL respectively, for TNF-α to differentiate the two groups. This demonstrates an ability to distinguish individuals with active IBD compared to healthy controls based on sweat measurements. To visualize and account for the temporal relationships of TNF-α obtained from the IBD AWARE device, readings collected from the sweat every 1 min were plotted for subjects with IBD and healthy controls (Fig. 4A). The mean continuous sweat TNF-α reading for subjects with active IBD (2.11 pg/mL) differed significantly from the mean value in healthy controls (0.19 pg/mL) (*p* < 0.0001) (Fig. 4B).

## Discussion

In this prospective observational study, we demonstrate that TNF-α can be measured continually in the sweat of subjects with IBD over multiple days. We found that sweat and serum TNF-α levels are correlated in subjects with active IBD. When compared to healthy controls, TNF-α levels in the sweat were found to be significantly higher in subjects with active IBD and differentiated the two groups. To the best of our knowledge this is the first study to passively assess longitudinal TNF-α in the sweat and the first to assess longitudinal sweat measurements in individuals with a chronic inflammatory disease over several days, utilizing a wearable device. This approach highlights a novel means to potentially monitor IBD and key markers of disease activity.

For this technology to be applied to chronic disease monitoring and to provide an advantage over point of care testing, such as serum C-reactive protein or fecal calprotectin measurements, further study is needed to understand the longitudinal relationship between sweat and serum-based markers. Our current study is the first application of a sweat-sensing wearable technology to the longitudinal monitoring of a chronic inflammatory disease and the first to evaluate the correlation between key sweat markers with serum levels when collected over multiple days. TNF-α is a model analyte to target in this pilot assessment given its role in the pathophysiology of IBD, as well as the differences in serum concentrations observed in those with and without IBD. Furthermore, anti-TNF-α therapy is a widely used and effective treatment for IBD. Baseline levels of serum TNF-α have been associated with treatment response in IBD and provide a good estimation of global disease burden and need for medication escalation in other chronic conditions, such as rheumatoid arthritis^37–39^. Thus, its continuous assessment in the monitoring of disease and treatment response, which is possible via sweat sensing wearables, warrants further evaluation. Importantly, our assessment demonstrated a linear relationship between sweat and serum TNF-α in subjects with active IBD, collected over a period of up to 5 days. This demonstrates that sweat analytes, collected by a wearable device, track serum values over time. Furthermore, as would be found in the serum, we demonstrated that subjects with active IBD had higher mean sweat TNF-α levels compared to healthy controls. These findings verify that concentrations of TNF-α in the sweat not only change over time in a similar manner to the serum but achieve different levels in accordance with expected differences between populations.

There are several limitations to our study. As this is a pilot study, the overall number of subjects is small, with only 16 subjects with IBD and 12 healthy controls participating. The 16 subjects with IBD were hospitalized as well during the study period. Therefore, further study is needed in a larger cohort of subjects and in outpatient populations. Our group is currently performing such studies and following subjects over longer periods of time. An additional limitation is that some subjects with IBD had serum measurements that were drawn at time points during which there were no overlapping sweat measurements obtained, which precludes the inclusion of these timepoints in the analysis. Last, there is only one serum measurement drawn each day. Additional serum measurements would have expanded our exploration of the relationship between continuously collected sweat levels with serum readings.

## Conclusions

In summary, we have demonstrated that the IBD AWARE sweat sensing wearable device, which senses passively expressed sweat in real time, is able to longitudinally monitor a cytokine in 16 subjects with IBD over a 5-day period. These readings are correlated with serum measurements. Furthermore, we demonstrated that sweat TNF-α levels in subjects with active IBD are elevated compared to healthy controls, which mirrors anticipated serum differences in these populations. This data demonstrates that sweat sensing wearable technology offers a possible novel means to monitor disease activity in a remote, passive, and real-time fashion and that further study is warranted.

## Materials and methods

### Study procedures

Two prospective cohorts of subjects, one with and one without IBD, were enrolled. We performed a prospective observational study enrolling subjects with IBD at the Mount Sinai Hospital in New York. Subjects were 18 years of age or older, diagnosed with IBD, admitted to the hospital for an IBD related admission and had an elevated C-reactive protein. Exclusion criteria included (1) a planned surgery, (2) active infection, or (3) a bowel obstruction. Healthy control subjects were enrolled at the University of Texas at Dallas, Dallas, Texas. They were at least 18 years of age and did not have an active infection or underlying chronic disease. This study was approved by both the institutional review board at Mount Sinai Hospital and the University of Texas at Dallas, respectively. Informed consent was obtained from all subjects.

Subjects with IBD who met inclusion criteria were enrolled and followed for up to 5 days during the index hospitalization. After signing the consent demographic information, medical history, and medication history were recorded. Throughout the index hospitalization medication usage was recorded. Daily labs were drawn that included TNF-α levels. Subjects provided clinical measures of disease activity each day. Subjects with CD completed a daily Harvey Bradshaw Index, while subjects with UC completed a Simple Clinical Colitis Activity Index. Subjects wore the IBD AWARE device in the antebrachial region of their arm throughout the study period. Subjects in the healthy control cohort wore the device for up to 48 h. Serum was not collected from the healthy controls. Data was synched from all subjects with the cloud server every 24 h and the device was replaced with a new charged one.

### Sweat sensing device and sweat processing

We have previously outlined the fabrication process for both the IBD AWARE device and its sensor. The sensor fabrication process has been adapted from Munje et al., and Jagannath et al., and has been described in detail previously^28,40^. In summary, the IBD AWARE device consists of a replaceable sweat-sensing strip specifically designed for target biomarkers. This strip is attached to a wearable electronic reader, which translates the sensor's impedance into a calibrated concentration of measured biomarker levels in sweat. Utilizing the screen-printing technique, we established a 2-electrode system that facilitates the transduction of the affinity-based interaction between the target biomarker and capture probe antibody, generating a measurable electrochemical signal. The schematic of the sensor device is depicted in the small top figure of Fig. 1. The sensor response was measured through non-faradaic electrochemical impedance spectroscopy (EIS). By applying a low sinusoidal input voltage, we recorded the resulting impedance, reflecting the binding of the target molecule to the capture probe antibody, at the frequency of 180 Hz. The sensing electrode was functionalized with a 10 mM thiol cross-linker. The opposite end of this cross-linker was bonded through aminolysis with 10 μg/mL of monoclonal TNF-α capture antibody to enable the specific detection of the target biomarker. The choice of a monoclonal antibody was deliberate, aiming to achieve a highly specific interaction^41,42^. The IBD AWARE device has undergone testing to assess its repeatability and stability in real world applications. This reproducibility analysis demonstrated the sensor’s ability to accurately measure sweat analyte levels over time, exhibiting a consistent response to spiked analyte concentrates with a coefficient of variation below 10% through a 4 week testing period^25,43^. Sweat TNF-α measurements were collected at a 1 min frequency.

### Statistical analysis

Analyses and figures were performed and created using GraphPad Prism version 9.3.0 software. Linear correlation coefficient was calculated using the serum to sweat ratios of all subjects with IBD, where the serum and sweat overlapped by at least 1 h. Each sweat value in this analysis was calculated by averaging sweat TNF-α measurements collected every 1 min over the 30 min window before and after each serum measurement. This window was chosen given the short half-life of TNF-α, which is less than 30 min^44^.

To compare sweat concentrations between subjects with and without IBD, the mean sweat TNF-α value was calculated for each subject. For subjects with IBD, a period of 30 min before and after each serum value was used. In healthy subjects this period was calculated 30–60 min after the device was applied in the morning, to match the time of day and period used in the IBD cohort. The mean, standard error, and 95% confidence intervals for the cohort of subjects with and without IBD was calculated. A t-test (α = 0.05) was used to determine whether the sweat TNF-α concentrations differed between subjects with and without IBD. Performance was assessed with the Area Under the Curve of the Receiver Operating Characteristic (auROC), sensitivity, and specificity. Given the frequent and nearly continuous measurements of sweat TNF-α obtained by the sweat sensing device, sweat TNF-α values were graphed for subjects with and without IBD, at the 1 min resolution of collection. The mean TNF-α levels were calculated for all subjects with and without IBD and compared using a t test (α = 0.05).

### Supplementary Information

Supplementary Tables.

## Data Availability

Data will be made available upon reasonable request to the corresponding author.
