# Supplementary material for: Longitudinal assessment of sweat-based TNF-alpha in inflammatory bowel disease using a wearable device
Source: Sci Rep. 2024 Feb 3;14:2833. doi: 10.1038/s41598-024-53522-1 (PMC10838338; doi:10.1038/s41598-024-53522-1)
Supplement: Supplementary file 1 — Supplementary Tables. [file 41598_2024_53522_MOESM1_ESM.docx]

Supplemental Table 1. Mean sweat TNF-α levels calculated over a +/-30-minute window around each serum TNF-α value. Participant values are included if a sweat measurement was available in this window. Clinical disease activity indices are provided for each subject and each day of assessment.

|  |  |  | TNF-α (pg/ml) | | Clinical Disease Activity Indices | |
| --- | --- | --- | --- | --- | --- | --- |
| Participant | Disease | Day | Sweat | Serum | SCCAI | HBI |
| 1 | CD | 1 | --- | --- |  | 10 |
|  |  | 2 | 1.74 | 9.58 |  | 9 |
|  |  | 3 | 2.48 | 13.66 |  | 4 |
| 2 | CD | 1 | --- | --- |  | 13 |
|  |  | 2 | 1.35 | 1.89 |  | 14 |
| 3 | CD | 1 | 1.91 | 10.13 |  | 1 |
|  |  | 2 | --- | --- |  | 1 |
|  |  | 3 | 2.04 | 9.04 |  | 3 |
|  |  |  |  |  |  | 2 |
| 4 | UC | 1 | 2.00 | 3.00 | 12 |  |
|  |  | 2 | 2.02 | 3.55 | 12 |  |
|  |  | 3 | 2.11 | 3.55 | 12 |  |
| 5 | UC | 1 | --- | --- | 7 |  |
|  |  | 2 | --- | --- | 5 |  |
|  |  | 3  4 | 0.31  1.10 | 0.15  0.36 | 4  8 |  |
| 6 | CD | 1 | 1.34 | 4.66 |  | 9 |
|  |  | 2 | 1.30 | 2.46 |  | 6 |
|  |  | 3  4  5 | 1.05  1.59 | 2.89  3.31 |  | 6  6  7 |
| 7 | UC | 1 | 0.58 | 2.04 | 5 |  |
|  |  | 2  3 | 0.51 | 2.46 | 5  4 |  |
| 8 | UC | 1 | 2.03 | 10.44 | 6 |  |
|  |  | 2 | --- | --- | 6 |  |
|  |  | 3  4  5 | 0.34  4.10 | 6.25  9.60 | 8  6  6 |  |
| 9 | UC | 1  2  3 | 1.61  0.88 | 0.36  0.36 | 9  6  5 |  |
| 10 | UC | 1  2  3  4 | ---  ---  1.86  1.93 | ---  ---  0.75  0.23 | 4  3  3  3 |  |
| 11 | UC | 1 | --- | --- | 5 |  |
|  | UC | 2  3 | 1.66 | 4.89 | 8  3 |  |
| 12 | UC | 1 | 1.88 | 5.15 | 5 |  |
|  | UC | 2 | 1.78 | 5.41 | 5 |  |
|  | UC | 3 | 1.53 | 8.26 | 4 |  |
|  | UC | 4  5 | 2.28 | 5.15 | 3  3 |  |

UC, ulcerative colitis; CD, Crohn’s disease; HBI, Harvey Bradshaw Index; SCCAI, Simple Clinical Colitis Activity Index; TNF, tumor necrosis factor.

Supplemental Table 2. Baseline demographic information for healthy controls.

|  | **Healthy Cohort, n=12 (%)** |
| --- | --- |
| Age, years, median | 28.5 |
| Sex |  |
| Female | 5 (41.7) |
| Male | 7 (58.3) |
| Race |  |
| White | 3 (25) |
| Black | 1 (8.3) |
| Asian | 8 (66.7) |
| Ethnicity |  |
| Not Hispanic | 12 (100) |
|  |  |
